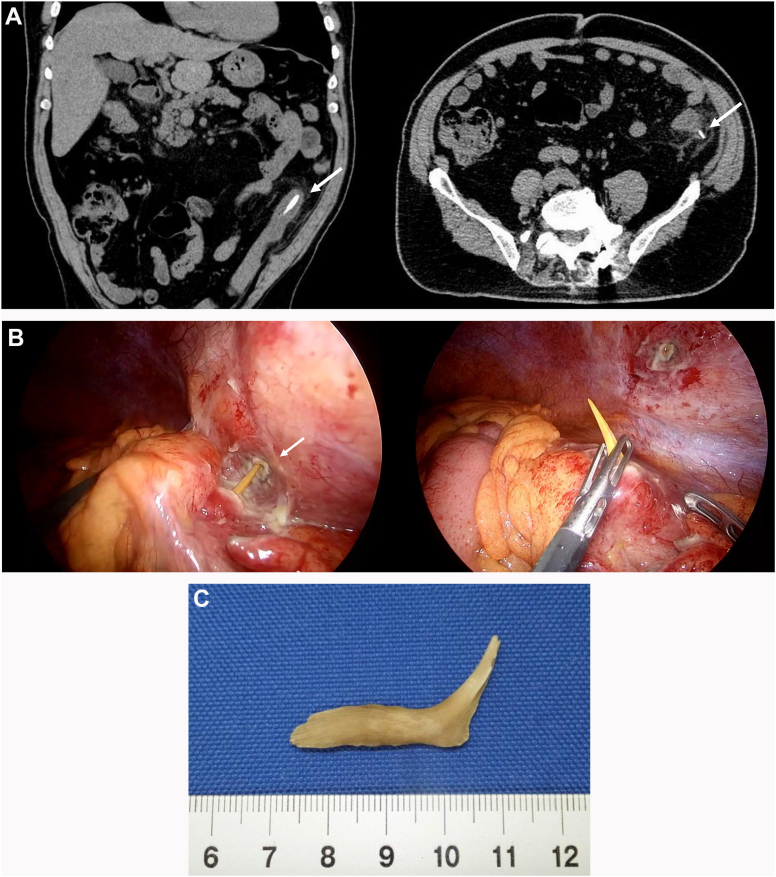# Fish Bone–Induced Descending Colon Perforation With Abdominal Wall Penetration

**DOI:** 10.1016/j.gastha.2026.101012

**Published:** 2026-05-18

**Authors:** Shigenori Masaki, Motoya Kashiyama

**Affiliations:** 1 Department of Surgery and Gastroenterology, Miyanomori Memorial Hospital, Sapporo, Hokkaido, Japan; 2 Department of Surgery, Kin-ikyo Chuo Hospital, Sapporo, Hokkaido, Japan

A 65-year-old man presented to the emergency department with a 3-day history of left-sided abdominal pain. Abdominal examination revealed severe tenderness and guarding in the left flank. Laboratory investigations showed markedly elevated inflammatory markers, with a C-reactive protein level of 20.59 mg/dL. Abdominal computed tomography demonstrated a linear hyperdense structure within the descending colon penetrating the colonic wall and extending into the abdominal wall (Figure A). The patient reported consuming salmon head soup 2 weeks earlier. Based on this history and imaging findings, fish bone–induced descending colon perforation was suspected. As the patient had already developed peritonitis, emergency laparoscopic surgery was performed. A fish bone penetrating the abdominal wall was identified and removed (Figure B). The perforation site was closed with sutures and reinforced with an omental patch. The extracted fish bone measured 4 cm in length and 8 mm in thickness (Figure C). The postoperative course was uneventful, and the patient was discharged 1 week later. Fish bone–induced perforation of the descending colon with penetration into the abdominal wall is extremely rare; prompt computed tomography diagnosis and surgical intervention can result in a favorable outcome.

**Figure:**